# Effects of Compound Probiotics on Production Performance, Apparent Digestion Rate of Nutrients and Serum Index of Pigs at Different Stages

**DOI:** 10.3390/ani16121877

**Published:** 2026-06-17

**Authors:** Haitao Chen, Yahui An, Hongzhan Cao, Chunlian Lu

**Affiliations:** College of Animal Science and Technology, Hebei Agricultural University, Baoding 071001, China; 13931938488@163.com (H.C.); 15511672602@163.com (Y.A.)

**Keywords:** compound probiotics, gestating sows, weaned piglets, growth performance, serum biochemical indices, immune indices

## Abstract

This experiment aimed to explore the effects of different doses of compound probiotics (a 1:1:1 mixture of *Saccharomyces cerevisiae*, *Lactobacillus acidophilus*, and *Bacillus subtilis*) added to the diet on pregnant sows and weaned piglets. The experiment was carried out in two stages. Experiment with pregnant sows: thirty-six second-parity Large White sows at 80 d of late gestation were randomly divided into a control group, experimental group I, and experimental group II. The control group was fed a basal diet, while experimental groups I and II were fed the basal diet supplemented with 2 g/kg and 3 g/kg of compound probiotics, respectively. The pre-experiment lasted 7 d, and the formal experiment continued until the end of lactation. The results showed that the numbers of live piglets per litter, healthy piglets per litter, litter birth weight and litter weaning weight in the experimental groups were significantly higher than those in the control group (*p* < 0.05). Colostrum IgG concentration in experimental group I was significantly higher than that in the control group and experimental group II (*p* < 0.05). Compound probiotics significantly increased colostrum immunoglobulin levels (*p* < 0.05). The concentrations of ammonia, carbon dioxide and PM2.5 in the barns of the experimental groups all showed a decreasing trend. Experiment with weaned piglets: a total of 160 Landrace × Yorkshire crossbred weaned piglets at 30 d of age with an initial body weight of (8.01 ± 0.13) kg were randomly assigned to four groups. The control group was fed a basal diet, while the treatment groups were supplemented with 2, 3, and 4 g/kg of compound probiotics, respectively. The results indicated that average daily gain and average daily feed intake in experimental group III were significantly higher than those in the control group, while the feed-to-gain ratio and diarrhea rate were significantly lower (*p* < 0.05). The apparent digestibility of crude fiber was significantly higher than that in the control group (*p* < 0.05), and serum IgA was significantly higher than in the other groups (*p* < 0.05). In conclusion, dietary supplementation with 2 g/kg compound probiotics for sows in late gestation showed the optimal effect, improving reproductive performance, colostrum immune indices and reducing harmful gases in the barn. For weaned piglets, supplementation with 4 g/kg compound probiotics improved growth performance, nutrient digestibility and serum immune indices.

## Simple Summary

This study aimed to explore the effects of dietary supplementation with different levels of compound probiotic preparations on production performance, immunity, and the breeding environment of gestating sows and weaned piglets. Feeding trials were conducted with probiotic supplementation at various doses. The results showed that adding 2 g/kg of compound probiotics to the diet of sows significantly improved reproductive performance and colostrum immune levels and reduced the concentrations of harmful gases and particulate matter in the pig housing. For weaned piglets, supplementation at 4 g/kg remarkably enhanced growth performance, apparent digestibility of crude fiber, and serum immune indicators, and also decreased the diarrhea rate. This study determined the optimal dosage of compound probiotics for different breeding stages of pigs. These findings indicate that compound probiotics can effectively enhance the reproductive performance of sows and the healthy growth of piglets and improve the breeding environment, providing an important scientific basis and practical support for the promotion and application of green and healthy pig production systems.

## 1. Introduction

With the promulgation of the policy banning antibiotics in feed, the comprehensive ban on antibiotics is not only a test for the feed industry but also an examination of the recent development of China’s animal husbandry industry. The ban on antibiotics in feed, reduction in antibiotics in breeding, and absence of antibiotics in products have become an irreversible development trend [1,2,3,4].

Europe’s ban on antibiotics in feed has brought growing pains to the livestock industry, accompanied by a rise in the incidence of certain diseases. While sales of therapeutic antibiotics have been on a long-term downward trend, they achieved two consecutive years of rebound growth in 2023 and 2024, and group medication remains the mainstream practice [5,6,7]. Establishing a virtuous cycle is the primary task after the feed antibiotic ban. Green breeding models, a sound breeding environment, and healthy and stable animal populations are the guarantees for a virtuous and healthy cycle. Breeding is a complex process, and exploring suitable solutions based on actual conditions is an inevitable path [8,9].

Probiotics and prebiotic preparations have been used as antibiotic alternatives in livestock and poultry breeding and have achieved good application effects in improving the growth performance of livestock and poultry [10,11]. As feasible microbial preparations, probiotics refer to a class of beneficial active microorganisms administered at an appropriate dose that alter the microbial community composition in a certain part of the host, serving as functional additives in modern dietary and agricultural practices [12]. *Lactic acid bacteria (LAB)* are centered around the *genus Lactobacillus*, which, together with the *genus Bifidobacterium*, represents the most widely commercialized and applied probiotics to date [13].

The beneficial effects of lactic acid bacteria on the host include enhancing immune function, inhibiting the adhesion of pathogens to epithelial surfaces, and improving digestion, which are associated with their ability to produce lactic acid and metabolites (e.g., antioxidants, organic acids, and antimicrobial compounds) [14]. In addition, various digestive enzymes produced by probiotics can decompose macromolecular nutrients in the diet and convert them into forms more easily absorbed by the intestinal tract, thereby improving the apparent nutrient digestibility of livestock and poultry [15,16].

Yang et al. [17] added *Lactobacillus* to the diet of piglets and found that *Lactobacillus* could improve the antioxidant status and immune function of piglets, induce the production of antimicrobial substances, and enhance the disease resistance of piglets, thereby promoting their better growth and reducing the incidence of diarrhea and mortality. Kostovova et al. [18] found that *Bifidobacterium*, *Lactobacillus*, and *Acidobacterium* isolated from the intestinal contents of wild boars could inhibit the growth of specific pathogens, produce exopolysaccharides and bacteriocins, and stimulate the production of interleukin-10, an anti-inflammatory cytokine, in porcine macrophages.

Studies have shown that adding compound probiotics to the diet of piglets increases the average daily gain (ADG) and decreases the feed-to-gain ratio (F/G) of piglets [19] and also improves the average daily feed intake (ADFI) and serum IgG levels of piglets [20]. Chen et al. [21] fed piglets with compound probiotics composed of *Lactobacillus*, *Bacillus subtilis*, and *Candida utilis* and found that the ADG and ADFI of piglets were significantly increased, and the serum IgM and IgG contents of piglets were also significantly elevated. Liu et al. [22] reported that sows fed with compound probiotics had significantly increased ADFI, litter weight at weaning, and serum contents of IgG, IL-6, and TNF-α.

Adding *Bacillus* (*Bacillus* spp.—ATCC PTA-6737, PTA-127113, and PTA-127114) to the diet can reduce weight loss and backfat loss of sows during lactation [23]; feeding compound probiotics (*L. johnsonii* CJ21 + *B. subtilis* BS15 + *B. licheniformis* BL21) to pregnant sows increases the number of live-born piglets and healthy piglets [24] and improves the average birth weight, average weaning weight, ADG and weaning survival rate of newborn piglets [25]. Feeding *Saccharomyces cerevisiae* var. *boulardii* CNCM I-1079 to piglets can significantly improve their growth rate, feed intake, feed efficiency and immune status [26]. However, the safety of probiotics has also drawn increasing attention, and the research literature on vaccines can serve as reference cases in clinical practice. Overall, the effects of probiotics are inconsistent. Existing studies have confirmed that maternal intake of probiotics during pregnancy may reduce infants’ vaccine immune responses in some cases. This highlights the importance of the timing of intake, routes of exposure and the developmental stages of infants and young children [27]. While certain probiotic or prebiotic treatments can bring significant benefits to intestinal health, feed conversion ratio and the yield of specific breeds or strains, other treatments may yield limited effects. Such varied responses may be attributed to genetic backgrounds, the initial composition of the human gut microbiome and environmental factors. In addition, young and healthy animals have different nutritional requirements from older or diseased ones [28].

These studies collectively demonstrate the important role of probiotics in pig production. At present, the application of probiotic preparations in the diets of weaned piglets and growing-finishing pigs is mostly focused on their effects on growth performance and early diarrhea, with few studies on serum immune and antioxidant indicators. In addition, there are few reports on the effects of the same compound probiotics on pigs at different growth stages. The application effect of the compound probiotics used in this experiment in pig production and its suitable addition level in the diets of pigs at different stages remain unclear. Therefore, this study aims to explore the dose effect of this compound probiotic in two critical breeding stages, namely late gestation sows and weaned piglets, determine the optimal supplemental dosage for each stage, and analyze its synergistic improvement effects on host production performance, immune function, nutrient utilization and barn environment. It intends to provide targeted intervention schemes with clarified strains and precise dosages for sustainable antibiotic-free pig farming.

## 2. Materials and Methods

### 2.1. Experimental Materials

This compound probiotic is formulated with *Saccharomyces cerevisiae*, *Lactobacillus acidophilus*, and *Bacillus subtilis* at a ratio of 1:1:1, with the total viable count not less than 1.1 × 10^9^ CFU/g. It should be stored at room temperature in a dark, dry and well-ventilated place. The strains used in this experiment were provided by Shijiazhuang Feilong Feed Co., Ltd. China (Baoding, China), in Hebei Province, and the product is named “Yikele”.

This series of compound probiotics has been in compliant mass production and on the market since 2017 (implemented in accordance with standard Q/01FL04—2017). The active strains in the Yikele compound probiotic formulation—*Saccharomyces cerevisiae*, *Bacillus subtilis*, and *Lactobacillus acidophilus*—are all listed in the permitted feed-grade microorganism catalog under Announcement No. 2045 of the Ministry of Agriculture of the People’s Republic of China (Feed Additive Variety Catalog [2013 Edition]). Specifically, *Saccharomyces cerevisiae* complies with GB 7300.501-2021 [29] (National Food Safety Standard—Feed Additives, Part 5: Microorganisms—*Saccharomyces cerevisiae*); *Bacillus subtilis* followed NY/T 2131-2012 [30] (Agricultural Industry Standard of the People’s Republic of China—Feed Additive: Bacillus subtilis) during the production period; viable cell enumeration employs GB/T 26428-2010 [31] (National Standard of the People’s Republic of China—Detection of *Bacillus subtilis* in Feed Microbial Additives); *Lactobacillus acidophilus* complies with GB 7300.504-2023 [32] (National Food Safety Standard—Feed Additives, Part 5: Microorganisms—*Lactobacillus acidophilus*), with testing conducted in reference to GB/T 20191-2025 [33] (National Standard of the People’s Republic of China—Microbiological Examination of *Lactobacillus acidophilus* in Feed); Product labeling and hygiene limits are implemented in accordance with GB 10648-2013 [34] (National Standard of the People’s Republic of China—Feed Label) and GB 13078 [35] (National Standard of the People’s Republic of China—Hygienical Standard for Feeds), respectively. The product standard number is Q/01FL 04-2023 (Enterprise Standard of Shijiazhuang Feilong Feed Co., Ltd.—Compound Probiotic Formulation).

### 2.2. Experimental Design and Grouping

#### 2.2.1. Experiment on Pregnant Sows

Thirty-six second-parity Yorkshire sows at the late gestation stage (80th day of gestation) were selected and randomly divided into a control group, experimental group I, and experimental group II, with 12 sows in each group (one sow per replicate, 12 replicates per group). The pretest period lasted 7 days, and the formal experimental period ranged from the 87th day of gestation to the end of lactation. The control group was fed a basal diet, while experimental groups I and II were fed the basal diet supplemented with compound probiotics at the levels of 2 g/kg and 3 g/kg, respectively.

#### 2.2.2. Experiment on Weaned Piglets

One hundred and sixty crossbred (Landrace × Yorkshire) weaned piglets with a similar body weight of (8.01 ± 0.13) kg and good health status were selected and randomly divided into a control group, experimental group I, experimental group II, and experimental group III, with 40 piglets in each group. Each group was further divided into 5 replicates with 8 piglets per replicate. The control group was fed a basal diet, while experimental group I, II, and III were fed the basal diet supplemented with compound probiotics at the levels of 2 g/kg, 3 g/kg, and 4 g/kg, respectively. The pretest period lasted for 7 days, and the formal test period lasted for 35 days.

### 2.3. Experimental Diets and Nutritional Levels

A reasonable experimental formula was formulated with reference to the nutritional levels recommended by the National Research Council of the United States (NRC 2012), and corn–soybean meal type diets were prepared. The detailed formulas and nutritional levels are shown in Table 1 and Table 2.

### 2.4. Feeding and Management

Pregnant sow group: Pregnant sows were housed in independent units, with each unit consisting of two pens and 6 sows per pen. The sows were fed three times daily, and their feed intake was gradually increased according to gestational stage. Seven days before farrowing, the sows were cleaned and then transferred to disinfected farrowing crates. Feeding was stopped 10 h prior to farrowing, and the farrowing process was monitored in real time while a quiet production environment was maintained. After farrowing, mucus on piglets was wiped off immediately, and special attention was paid to piglet thermal insulation.

Weaned piglet group: The pig house was thoroughly cleaned and disinfected before the trial commenced. After weaning, piglets were weighed, ear-tagged and moved to the disinfected pig house. They were fed wet feed with ad libitum access to feed and water.

### 2.5. Sample Collection and Processing

#### 2.5.1. Collection of Colostrum Samples

Milk secreted by sows within 3 days after farrowing was defined as colostrum. In the experiment, colostrum collection was completed within 2 h after the onset of farrowing for each sow, with 4 sows selected for sampling in each group. Colostrum was collected by gently squeezing the base of the nipples into sterile containers, then aliquoted into 20 mL sterile Eppendorf tubes (EP) tubes and stored at −20 °C for subsequent analysis.

#### 2.5.2. Feed and Fecal Samples

Feed samples: After the preparation of experimental diets, approximately 300 g of feed was randomly collected from each group as a test sample and stored in a low-temperature and dry environment for subsequent analysis of conventional nutrient contents.

Fecal sample collection: During the last 3 days of the formal test period, 2 pigs were randomly selected from each replicate for continuous fecal collection. Fresh fecal samples were collected in the morning, noon and evening every day, and the daily samples from each pig were mixed thoroughly. A subsample accounting for 2–10% of the total weight was taken, and 0.1 mL of 10% hydrochloric acid was added per gram of feces for nitrogen fixation. All samples were hermetically sealed and stored at −20 °C for subsequent analysis. Processing: For the determination of crude protein (CP) content, the preserved fecal samples were dried in a blast drying oven at 65 °C for 48 h, equilibrated to ambient moisture for 12 h, and then ground and sieved through 10-mesh and 40-mesh sieves. For the determination of crude fiber, crude fat and other indicators, fresh fecal samples without hydrochloric acid addition were directly dried in an oven at 65 °C for 48 h and equilibrated to ambient moisture for 12 h.

#### 2.5.3. Blood Collection and Serum Preparation

At the end of the experiment and before weighing, 5 pigs per group were randomly selected from each group and fasted for 12 h but allowed free access to water. Blood samples were collected from the anterior vena cava, allowed to stand at room temperature for 10 min, and then centrifuged at 3000 rpm for 10 min to separate serum. The serum samples were stored at −20 °C for subsequent analysis of serum biochemical parameters.

### 2.6. Determination Indicators and Methods

#### 2.6.1. Performance of Pregnant Sows

Based on the farrowing outcomes, the birth weight of piglets (weighed immediately after wiping off mucus on the piglets’ body surface), number of live born piglets per litter, number of healthy born piglets per litter (piglets with a body weight less than 800 g were defined as weak piglets), total litter birth weight and total litter weaning weight were recorded and statistically analyzed. The birth weight of individual piglets and total birth weight per litter were measured within 12 h after farrowing.

#### 2.6.2. Growth Performance

Average daily feed intake (ADFI) was calculated based on the recorded daily feed supply and residual feed amount; average daily gain (ADG) was determined by recording the initial body weight before the experiment, the fasting body weight at the end of the experiment, and trial duration. The feed-to-gain ratio (F/G) was calculated as the ratio of total feed consumption to total body weight gain of experimental pigs during the trial. The formulas are as follows:

Average daily feed intake (ADFI) (g/d) = (Feed supply amount − Residual feed amount)/Trial days

Average daily gain (ADG) (g/d) = (Final body weight of experimental pigs − Initial body weight of experimental pigs)/Trial days

Feed-to-gain ratio (F/G) = Total feed consumption during the trial/Total body weight gain of experimental pigs.

#### 2.6.3. Determination of Apparent Nutrient Digestibility

The apparent digestibility of crude protein, crude fat, crude fiber, gross energy, and calcium in feed samples was determined, referring to the 4th edition of Feed Analysis and Feed Quality Inspection Technology. The calculation formula for nutrient apparent digestibility is presented as follows:

Apparent digestibility of a certain nutrient (%) = 100 × [1 − (A/B) × (C/D)].

where A is the content of a certain nutrient in fecal samples (%); B is the content of a certain nutrient in feed (%); C is the content of acid-insoluble ash in feed (%); and D is the content of acid-insoluble ash in fecal samples (%).

#### 2.6.4. Determination of Serum Indicators

Determinations were performed using a microplate reader (ELX-808, BioTek Instruments, Inc., Winooski, VT, USA) and a semi-automatic biochemical analyzer (HITACHI7070, Hitachi, Ltd., Tokyo, Japan). The detected indices included alkaline phosphatase (AKP), aspartate aminotransferase (AST), alanine aminotransferase (ALT), calcium, phosphorus, blood urea nitrogen (BUN), albumin (ALB), total protein (TP), and total cholesterol (T-CHOL). Serum antioxidant indices: malondialdehyde (MDA), superoxide dismutase (SOD), and total antioxidant capacity (T-AOC). Enzyme-linked immunosorbent assay (ELISA) was adopted to determine serum immune indices, including immunoglobulin A (IgA), immunoglobulin M (IgM), immunoglobulin G (IgG), interleukin-2 (IL-2), interleukin-4 (IL-4), complement 3 (C3), and complement 4 (C4). All reagents used for the above determinations were purchased from Nanjing Jiancheng Bioengineering Institute, Nanjing, China.

#### 2.6.5. Determination of Air Quality Indicators in Pig Houses

Air quality in pig houses was monitored, including the harmful gases ammonia (NH_3_), hydrogen sulfide (H_2_S), carbon dioxide (CO_2_), as well as PM2.5 and PM10. Six detection sites were selected according to the structure of the pig houses. Data collection for indicators such as CO_2_, NH_3_, H_2_S, PM2.5 and PM10 was conducted at four time points (7:00, 11:00, 14:00 and 18:00) every day for continuous monitoring. A portable integrated gas detector (BH-4A) was used for detection, with the original output unit of ppm. Pollutant concentrations were expressed as mass-volume concentration (mg/m^3^), representing the pollutant mass per cubic meter of air. The conversion formula between mg/m^3^ and ppm is presented as follows:

X = M/22.4·ppm·[273/(273 + T)] × (Ba/101,325)

where X—concentration of a certain harmful gas in milligrams per standard cubic meter; ppm—measured volume concentration value; M—molecular mass of a certain harmful gas; T—temperature; and Ba—pressure

### 2.7. Statistical Analysis

Experimental data were sorted using Excel 2016, while SPSS 22.0 was applied for statistical analyses including the Shapiro–Wilk normality test and Levene’s test for homogeneity of variance. Data satisfying normal distribution and homogeneity of variance were subjected to one-way ANOVA for intergroup comparisons, followed by the LSD test for multiple pairwise comparisons. Results were expressed as mean ± standard deviation, and statistical significance was defined at *p* < 0.05.

## 3. Results

### 3.1. Effects of Different Addition Levels of Compound Probiotics on Pregnant Sows

#### 3.1.1. Effects of Compound Probiotics on the Production Performance of Pregnant Sows

As shown in Table 3, the average birth weight of piglets in the control group was significantly higher than that in the experimental groups (*p* < 0.05), being 11.20% and 16.81% higher than that in experimental groups I and II, respectively (*p* < 0.05). The number of live-born piglets per litter in experimental groups I and II increased by 17.37% and 20.07% compared with the control group, with statistically significant differences (*p* < 0.05). The number of viable piglets in experimental group I was significantly higher than that in the other two groups. Additionally, the litter birth weight in experimental group I was significantly higher than that in the other two groups. The litter weaning weight of piglets in the experimental groups was also significantly higher than that in the control group (*p* < 0.05).

#### 3.1.2. Effects of Compound Probiotics on Colostrum Immune Indicators in Lactating Sows

As shown in Table 4, the colostrum IgG level in experimental group I was significantly higher than that in the other two groups; the IgG levels in experimental group I and experimental group II were both higher than that of the control group, with significant differences observed among all three groups (*p* < 0.05). The IgM level in colostrum increased with the rising addition level of compound probiotics, reaching the peak at the addition level of 3 g/kg, and there were significant differences among the three groups (*p* < 0.05). Compared with the basal control group, the IgM level in colostrum of experimental group II was increased by 28.60%. Meanwhile, the IgA level was also the highest in experimental group II, which was significantly higher than that of the other two groups, while experimental group I had the lowest IgA level with a significant difference (*p* < 0.05).

#### 3.1.3. Effects of Compound Probiotics on Air Indicators in Pregnant Sow Houses

As shown in Table 5, the indoor NH_3_ concentration of pregnant sow houses in the experimental groups was significantly lower than that in the control group (*p* < 0.05). No significant difference in H_2_S concentration was observed between the experimental groups and the control group (*p* > 0.05). The CO_2_ concentration showed a decreasing trend, among which experimental group II had a significantly lower CO_2_ concentration than the control group and experimental group I (*p* < 0.05). The PM2.5 concentration in experimental group II was significantly reduced compared with the control group (*p* < 0.05).

### 3.2. Effects of Different Addition Levels of Compound Probiotics on Weaned Piglets

#### 3.2.1. Effects of Compound Probiotics on Growth Performance in Weaned Piglets

As shown in Table 6, the weaned piglets in experimental group III supplemented with 4 g/kg compound probiotics exhibited significantly higher average daily gain (ADG) and average daily feed intake (ADFI) than those in the control group (*p* < 0.05). The feed-to-gain ratio differed significantly between the control group and experimental groups II and III (*p* < 0.05), with experimental group III which had the lowest feed-to-gain ratio. The diarrhea rate decreased with increasing probiotic supplementation; experimental group III had a significantly lower diarrhea rate than the control group (*p* < 0.05).

#### 3.2.2. Effects of Compound Probiotics on Apparent Nutrient Digestibility of Weaned Piglets

As shown in Table 7, the apparent digestibility of crude protein (CP), ether extract (EE) and calcium (Ca) in weaned piglets was positively correlated with the supplemental level of compound probiotics, with no significant differences observed among all groups (*p* > 0.05). The crude fiber (CF) digestibility in the experimental groups was significantly higher than that in the control group (*p* < 0.05). The gross energy (GE) digestibility in experimental group II and experimental group III was significantly higher than that in the other groups (*p* < 0.05).

#### 3.2.3. Effects of Compound Probiotics on Serum Biochemical Indicators of Weaned Piglets

As shown in Table 8, significant differences in AST levels were observed among the control group, experimental group I, experimental group II, and experimental group III (*p* < 0.05). Experimental group II had the lowest ALT level, significantly lower than that in the other three groups (*p* < 0.05), while no significant difference was detected among the remaining groups. The serum phosphorus level was highest in experimental group II, differing significantly from that in experimental group I (*p* < 0.05), but not from the control group or experimental group III. Serum calcium level was significantly lower in experimental group I than in the other three groups (*p* < 0.05). The control group exhibited the highest BUN level, which was significantly higher than that in experimental groups II and III (*p* < 0.05).

#### 3.2.4. Effects of Compound Probiotics on Serum Antioxidant Indicators of Weaned Piglets

The serum antioxidant indices of weaned piglets are presented in Table 9. The serum T-AOC level was the highest in experimental group III and the lowest in the control group, with no significant differences among all groups (*p* > 0.05). The serum MDA level peaked in experimental group I and was the lowest in experimental group II, with no significant difference detected among groups (*p* > 0.05). The serum SOD content in the control group was significantly lower than that in the other three groups (*p* < 0.05), whereas no significant difference was found among the three experimental groups (*p* > 0.05).

#### 3.2.5. Effects of Compound Probiotics on Serum Immune Indicators of Weaned Piglets

As shown in Table 10, the IgG level was the highest in experimental group III and the lowest in experimental group I, with a significant difference between the two groups (*p* < 0.05), while no significant differences were observed between the control group and other groups (*p* > 0.05). The serum IgA level in experimental group III was the highest, which was significantly higher than that in the control group, experimental group I, and experimental group II (*p* < 0.05). The IL-2 level reached the peak in experimental group III, with no significant differences among all groups (*p* > 0.05). The IL-4 level was the highest in experimental group II, with no significant differences detected among all groups (*p* > 0.05).

## 4. Discussion

### 4.1. Effects of Compound Probiotics on Pregnant Sows

#### 4.1.1. Analysis of Production-Regulating Effects of Compound Probiotics in Pregnant Sows

Fetal development during late gestation causes uterine compression on the rectum, which inhibits intestinal peristalsis, induces intestinal flora imbalance, and increases rectal temperature, thereby adversely affecting the uterine microenvironment. Dietary supplementation of compound probiotics can optimize intestinal ecological balance, ameliorate flora dysbiosis, stimulate intestinal peristalsis, and improve nutrient digestion and absorption capacity. These improvements provide sufficient nutrients for fetal growth, guarantee normal embryonic development, and ultimately enhance sow reproductive performance [36,37]. From day 80 of gestation to farrowing, fetal weight gain accounts for approximately 71% of the final piglet birth weight; therefore, the nutritional status of sows during this critical stage is essential for litter performance [38,39,40]. In the present study, dietary compound probiotics supplementation was initiated on day 80 of gestation. The results indicated that the 2 g/kg supplementation level reduced piglet birth weight compared with the control group but improved litter uniformity. Although extremely low birth weight usually reduces piglet survival rate, piglets with slightly lower birth weight and higher litter uniformity can fully realize their growth potential via rational nutritional supply during the lactation and nursery periods [41]. Meanwhile, experimental groups exhibited a significantly higher number of live-born piglets per litter than the control group in the present study. Ma et al. reported that dietary supplementation with fermented liquid containing *Lactobacillus plantarum B90* (CGMCC1.12934) and *Saccharomyces cerevisiae P11* (CGMCC2.3854) significantly increased the individual weight of 1-day-old piglets [42], which was consistent with the variation trend observed in this experiment. Chen et al. found that dietary supplementation with 2% yeast hydrolysate markedly improved total litter weight, average piglet weight, and average daily gain of suckling piglets at days 7 and 19 of lactation [43]. These findings support the conclusion that yeast-derived probiotic products can promote lactational feed intake and improve piglet growth performance [44]. The relatively lower individual piglet birth weight in the probiotic-supplemented groups in this study indicated that compound probiotics might regulate maternal nutrient distribution and placental nutrient transport efficiency in sows [45,46]. Specifically, probiotic intervention could balance fetal intrauterine growth within the same litter and improve litter uniformity, which may reduce individual fetal birth weight to a certain extent.

#### 4.1.2. Analysis of Immunomodulatory Effects of Compound Probiotics on Colostrum Indicators in Lactating Sows

Probiotics (especially lactic acid bacteria and bacilli) may be taken up by microfold cells (M cells) in the intestinal Peyer’s patches and then presented to dendritic cells (DCs) and B cells. This further activates the T follicular helper cell (Tfh)-dependent IgM-to-IgA class switch recombination (CSR) pathway, thereby enhancing immunity and exerting an effect on the immune indicators in colostrum [47]. Since the nutritional requirements and immunity of suckling piglets are entirely derived from sows, the health status of sows directly affects the growth performance of piglets [48]. Therefore, early access to colostrum is critical for acquiring passive immunity. Colostrum is characterized by a high concentration of immunoglobulins, mainly IgG. In this experiment, when the addition level of compound probiotics was 2 g/kg, the IgG content in colostrum was significantly higher than that in the control group. The IgG level in colostrum and the amount of colostrum ingested by piglets determines the amount of IgG obtained by piglets [49]. Compared with IgG, IgM is more potent in terms of lytic, bactericidal, agglutinating, and complement-activating activities. Although its content is low, it plays a crucial role in early immune defense. IgA accounts for 10–20% of the total serum immunoglobulins, second only to IgG. Secretory IgA (SIgA) is the main component of the body’s mucosal defense system, and the IgA in colostrum is secretory IgA [50]. In this experiment, the IgA content in colostrum was the highest when the addition level of compound probiotics was 3 g/kg. Ye found that supplementing the diet of pregnant sows with probiotics had no significant effect on the immunoglobulin levels in colostrum [51]; however, the immunoglobulin content showed an increasing trend. Tian reported that supplementing the diet of pregnant sows with probiotic fermented feed led to an increasing trend in all immunoglobulins compared with the blank group [52], with significant increases in IgG and IgM. Meanwhile, the birth litter weight and weaning litter weight were both higher than those in the control group, which was consistent with the results of this experiment. In this study, compound probiotics significantly increased the levels of IgG and IgM in sow colostrum. However, the level of IgA in experimental group I was lower than that in the control group. This may be because low-dose compound probiotics tend to activate Th2/humoral immunity, promote B cell differentiation and the production of IgG and IgM, yet provide insufficient stimulation for the class switch recombination (CSR) of IgA, or because they contain inadequate strains that induce the TGF-β pathway.

#### 4.1.3. Analysis of Air Quality-Regulating Effects of Compound Probiotics in the Housing of Pregnant Sows

The main harmful gases in pig housing include CO_2_, NH_3_, and H_2_S, among others. An increase in the concentration of harmful gases leads to a decline in indoor air quality, which in turn affects the health and production performance of pigs. Harmful gases in pig housing are mainly derived from feces and urine [53]. A study [54] found that dietary probiotic supplements can improve protein absorption and utilization efficiency, with significant effects observed at 4 h, 12 h and 24 h, also reducing fecal nitrogen content, which can effectively reduce NH_3_ production in the housing. This indicates that feeding compound probiotics to pigs may improve nitrogen digestibility and thereby help reduce NH_3_ emissions. This is likely because the microbial utilization of protein in the intestines is enhanced, which boosts nitrogen conversion efficiency and alleviates the burden on the urea cycle [55]. According to Environmental Parameters and Environmental Management for Standardized Pig Farms (GB/T 17824.3-2008) [56], the concentration of NH_3_ in pregnant sow housing should not exceed 25 mg/m^3^. In this experiment, the ammonia (NH_3_) concentration in the housing of the control group was 19.73 ± 1.59 mg/m^3^. It decreased to 14.44 ± 1.20 mg/m^3^ in the group supplemented with 2 g/kg probiotics and further dropped to 12.92 ± 1.76 mg/m^3^ in the 3 g/kg group. Additionally, the ammonia concentration showed a declining trend as the addition amount of compound probiotics increased. The underlying mechanism may be that compound probiotics can improve the protein digestion of pregnant sows [22]. Chen found that when the concentration of H_2_S in the housing is higher than 10 mg/m^3^ [57], the growth performance and nutrient digestibility of piglets are reduced. In this experiment, the H_2_S concentrations in all three groups were relatively low, which ruled out the influence of H_2_S on the production performance of pregnant sows.

### 4.2. Effects of Compound Probiotics on Weaned Piglets

#### 4.2.1. Analysis of Growth-Promoting Effects of Compound Probiotics in Weaned Piglets

The gastrointestinal tract of piglets is underdeveloped, and environmental and dietary changes after weaning can induce stress, leading to reduced gastrointestinal digestive enzyme activity and decreased absorption capacity, as well as malabsorptive diarrhea. Therefore, reducing stress factors and rational nutrition formulation are key to maintaining the growth performance of piglets [58,59]. Compound probiotics help maintain gut microbiota balance and produce antimicrobial substances (hydrogen peroxide and bacteriocins, among others), thereby reducing the incidence of bacterial diarrhea. Lin et al. [60] added different levels of probiotic preparations to weaned piglets and found that 0.15% supplementation resulted in the most significant reduction in diarrhea incidence relative to other experimental groups, which is consistent with the findings of Huang [61]. In this experiment, the diarrhea rate of experimental group III (4 g/kg) was reduced by 30% compared with experimental group I (2 g/kg), and diarrhea severity was significantly reduced, indicating that the supplementation level of compound probiotics is an important factor affecting diarrhea alleviation. In this experiment, when the supplementation level was 4 g/kg, the average daily gain (ADG) of weaned piglets was significantly higher than that of the control group, and ADG was positively correlated with the supplementation level of compound probiotics. When the supplementation level was 2 g/kg in the diet of weaned piglets, there was no effect on ADG compared with the control group. This may be because opportunistic pathogens such as *Escherichia coli* in the intestine of weaned piglets multiplied rapidly after weaning. The low dosage of compound probiotics resulted in an insufficient number of probiotics reaching the intestine, which put them at a disadvantage in the competition for ecological niches [21]. The feed-to-gain ratio is a direct reflection of feed conversion efficiency and a comprehensive reflection of nutrient digestibility. The feed conversion efficiency of the experimental group was higher than that of the control group, which was consistent with the findings of Xie et al. [62] and Yang [63]. This improvement in growth performance may be attributed to enhanced intestinal morphology. It significantly increases the villus height of the jejunum and ileum as well as the ratio of villus height to crypt depth, which may help enhance feed digestion and nutrient absorption [62]. The experiment concluded that the suitable supplementation level of compound probiotics is 4 g/kg, which also indicates that the antibiotic-free basal diet has a great impact on the growth performance of pigs in actual production. Meanwhile, the comparison of production performance shows that the growth performance advantage of the experimental groups was further enhanced compared with the control group.

#### 4.2.2. Analysis of Regulatory Effects of Compound Probiotics on Apparent Nutrient Digestibility in Weaned Piglets

The strains in probiotic preparations can produce various digestive enzymes, vitamins and other components in the gastrointestinal tract; digestive enzymes can promote the decomposition of nutrients and help improve the host’s absorption of nutrients. Compound probiotics utilize the synergy and antagonism of different strains, which have a better effect on promoting the formation of intestinal microflora, thereby improving the digestion and absorption of nutrients [64]. In the weaned piglet experiment, the apparent digestibility of crude protein (CP) and gross energy (GE) was highest when the supplementation level of compound probiotics was 4 g/kg, indicating that supplementation level is a key factor affecting nutrient digestibility. Huang added different levels of *Bacillus subtilis* and found that CP digestibility in the *B. subtilis* groups was higher than that in the control group, peaking at the 0.2% supplementation level (medium-level group) [61], and the CP digestibility was the highest when the supplementation level was 0.2% (the medium-level group), which indicated that a higher addition level is not always better, and this is consistent with the results of this experiment. In this experiment, the apparent digestibility of crude fiber (CF) in the experimental groups was significantly higher than that in the control group, which was related to the enzymes secreted by *Bacillus subtilis* in compound probiotics. Hou et al. [65] confirmed that the addition of *Bacillus quercus* can accelerate cellulose degradation and disrupt the hemicellulose structure in the cell wall during fermentation, thus improving the apparent digestibility of CF in the diet. Therefore, the advantage of the experimental groups in production performance was further enhanced.

#### 4.2.3. Analysis of Metabolic-Regulating Effects of Compound Probiotics in Weaned Piglets and Growing-Finishing Pigs

Serum biochemical indicators are important references for evaluating the health status and metabolic level of animals. The activities of aspartate aminotransferase (AST) and alanine aminotransferase (ALT) are indicators for assessing liver status, and the activities of AST and ALT may decrease when excessive fat accumulates in the liver [66]. The AST activities in experimental groups I and III of weaned piglets were higher than those in the control group, while ALT activity showed a decreasing trend. Clinically, the simultaneous increase in AST and ALT can reflect liver damage, and the increase in AST and decrease in ALT in the weaned piglet experiment do not affect the evaluation of liver status, which is consistent with the variation pattern of AST and ALT in the experiment of compound microecological preparations on weaned piglets by Ran et al. [67]. Total cholesterol (T-CHOL) can reflect the development of adipose tissue and fat deposition capacity, and a decrease in its content indicates weakened fat deposition capacity. Blood urea nitrogen (BUN) can reflect the efficiency of protein metabolism and absorption in the body. The content of total protein (TP) directly reflects the nutritional status, growth and development of animals, as well as the protein metabolism in the body. Albumin (ALB) can maintain colloid osmotic pressure and is also the main transport protein in plasma [68]. In the experiment, compound probiotics had no significant effect on the serum levels of TP and ALB, and the TP index did not increase with the increase in ALB level, which was consistent with the trend reported by Zhu [69]. The different trend from Wang’s experimental results may be caused by the difference in globulin [70]. In this experiment, the BUN level showed a decreasing trend. Generally, an acute increase in BUN level reflects renal insufficiency, and a low level often reflects damage to liver and kidney functions. Although there were significant differences in BUN levels among all groups in the weaned piglet experiment, the levels were within the normal reference range. The synergistic regulatory effect of compound probiotics on serum biochemical indicators needs further research.

#### 4.2.4. Analysis of Antioxidant-Regulating Effects of Compound Probiotics in Weaned Piglets

Weaning is one of the stages with the most severe oxidative stress during the life cycle of piglets. Studies have shown that from the 3rd to the 7th day after weaning, the activities of superoxide dismutase (SOD) and glutathione peroxidase (GSH-Px) in the jejunal mucosa of piglets decrease significantly, while the content of malondialdehyde (MDA) rises markedly. Meanwhile, the content of mitochondrial DNA and the activity of oxidative phosphorylation complexes are reduced, and the expression of tight junction proteins (occludin, claudin-1, and ZO-1) is down-regulated [71]. Claudins play a key role in maintaining the normal tissue morphology of the intestinal tract and promoting the integrity and permeability of the intestinal barrier [72]. This redox imbalance not only directly damages cell membrane lipids but also exacerbates inflammatory responses by impairing the integrity of the intestinal barrier, thus forming a vicious cycle of “oxidative stress-inflammation-barrier damage”. Free radicals have strong oxidizing properties, and the free radicals in the body are in a dynamic balance after metabolism. Nutritional interventions can enhance the body’s ability to scavenge free radicals and boost its antioxidant capacity [73]. Antioxidant capacity is closely associated with health status. Antioxidation refers to inhibiting the production of free radicals and eliminating the already generated ones, which can be achieved either by acting directly on free radicals or by indirectly consuming substances that are prone to generating free radicals [74]. *Lactic acid bacteria* in compound probiotics can produce active antioxidant substances such as antioxidant enzymes and manganese ions, thereby improving the antioxidant capacity of animals. The results of this experiment indicated that dietary supplementation with compound probiotics could increase the total antioxidant capacity (T-AOC) level of the experimental pigs, which was related to the activation of the Nrf2 pathway and the up-regulation of the expression of downstream antioxidant enzymes including catalase (CAT), superoxide dismutase (SOD), and glutathione peroxidase (GSH-Px) [75]. The malondialdehyde (MDA) levels showed a decreasing trend in both weaned piglets and growing-finishing pigs, which is basically consistent with the findings of Ma et al. [76]. In this experiment, SOD activity showed a decreasing trend in both growth stages. Smallfield et al. [77] suggested that the bacterial concentration might act as a physiological stressor, yet the underlying mechanism of this reaction remains unclear and requires further in-depth investigation. These results suggest that compound probiotics can improve the total antioxidant capacity in the serum of experimental pigs at both stages, as reflected by the changes in MDA levels.

#### 4.2.5. Analysis of Immunomodulatory Effects of Compound Probiotics in Weaned Piglets

Serum immunoglobulin levels are important indicators for evaluating the humoral immune status of the body, and they are generally measured by testing IgG, IgA, and IgM. Elevated levels of these immunoglobulins typically indicate an active inflammatory immune response. Compound probiotics may enhance the local synthesis of secretory immunoglobulin A (sIgA) in the intestinal tract; sIgA is secreted by plasma cells in the intestinal lamina propria, which differentiate from B cells in Peyer’s patches. A small fraction of IgA is transported across the epithelium into the circulatory system, leading to an increase in serum IgA levels [78]. In the weaned piglet experiment, there were no significant differences in the levels of IgM and IgG between the control group and the experimental groups, but the IgG level of experimental group III showed an increasing trend compared with the control group. The IgA level of experimental group III was significantly higher than that of the control group, which might be attributed to Bacillus subtilis in the compound probiotics binding to lymphoid antigen-binding sites in the intestinal tract and thereby increasing the number of T cells and B cells, leading to elevated IgA levels [79]. However, whether the elevated serum IgA truly reflects the strengthening of the intestinal mucosal immune barrier remains to be verified by measuring fecal sIgA concentrations or the number of IgA^+^ plasma cells in the intestine. In addition, the insignificant changes in IgG and IgM may be related to strain composition, the dosage threshold or the developmental stage of the host’s immunity, suggesting that the immunomodulatory effect of compound probiotics is not a “comprehensive enhancement”, but rather selective and condition-dependent. In the weaned piglet experiment, compound probiotics also had no significant effect on complement proteins or cellular inflammatory factors.

## 5. Conclusions

This study reveals the stage-specific dosage strategy and immune polarization effect of compound probiotics in swine production:

Pregnant Sows: A dosage of 2 g/kg is optimal for IgG production, balancing reproductive efficiency and environmental benefits; a 3 g/kg dosage shifts to trigger IgM/IgA activation and markedly improves barn air quality while attenuating the IgG-boosting effect. Such dosage-isotype specificity offers a novel approach for precise regulation of colostrum immune quality.

Weaned piglets: A total of 4 g/kg serves as the sole fully effective dosage that delivers improvements in growth performance, digestive function, physical health and immunity; 2 g/kg shows no overall efficacy and is confirmed below the minimum effective dosage; and 3 g/kg induces partial activation, resulting in improved growth but suboptimal health conditions.

The dose–effect relationship of probiotics is not linear; rather, it is comprehensively modulated by host developmental stage, intestinal microecological state, immune maturation, and physiological stress level. Future research should move beyond the empirical mindset of “higher dosage yields better outcomes” to establish precise dosage optimization models based on organ receptor saturation, systemic metabolites, and immune regulatory pathways, facilitating the practical application of probiotics in commercial swine production.

## Figures and Tables

**Table 1:** Feed formula and nutritional level of sows (%).

Items	Group	
	Latter Half of Gestation	Suckling Period, Age of Sucking, Lactation
Ingredients		
Corn	61.00	61.00
Wheat bran	13.00	10.00
Soybean meal	14.00	17.00
Fish meal	0.80	1.30
Expanded soybean	6.30	6.00
Limestone	0.80	0.60
CaHPO_4_ (calcium hydrogen phosphate)	1.50	1.50
NaCl (sodium chloride)	0.50	0.50
Soybean oil	0.80	0.80
Lys (lysine)	0.30	0.30
Premix ^1^	1.00	1.00
Total	100.00	100.00
DE (digestible energy)/(kcal/kg) ^2^	3250	3420
CP (crude protein) %	14.50	16.80
Lys (lysine) %	0.70	0.85
CF (crude fiber) %	4.80	4.50
Ca (calcium) %	0.75	0.85
P (phosphorus) %	0.55	0.55

Note ^1^: The premix provides: per kilogram of feed. VB1 1 mg; VB2 2.3 mg; VB6 1.5 mg; VB12 0.01 mg; VA 1160 IU; VD 178 IU; VE 10 IU; VK3 2 mg; nicotinic acid 12 mg; pantothenic acid 10 mg; folic acid 0.5 mg; biotin 0.09 mg; choline chloride 300 mg; copper 4 mg; zinc 53 mg; ferrum 53 mg; iodine 0.12 mg; manganese 2 mg; selenium 0.13 mg. Note ^2^: Digestible energy and lysine are calculated values (derived from nutrient content per ingredient in the nutrition facts table × ingredient ratio), while the remaining values are measured.

**Table 2:** Dietary formula and nutritional level of weaned piglets (%).

Items	Group
	Weaned Piglet Group
Ingredients	
Corn	65.00
Wheat bran	1.40
Soybean meal	11.00
Expanded soybean	6.00
Fish meal	2.00
Fermented soybean meal	4.00
Soy protein concentrate	2.50
Limestone	0.80
Whey powder	2.50
CaHPO4 (calcium hydrogen phosphate)	1.00
NaCl (sodium chloride)	0.30
Soybean oil	2.00
Lys (lysine)	0.50
Premix ^1^	1.00
Total	100.00
DE (digestible energy)/(kcal/kg) ^2^	3520
CP (crude protein) %	18.20
Lys (lysine) %	1.34
CF (crude fiber) %	2.50
Ca (calcium) %	0.65
P (phosphorus) %	0.55

Note ^1^: The premix provides: per kilogram of feed. VB1 1 mg; VB2 2 mg; VB6 1 mg; VB12 0.01; VA 1300 IU; VD 3160 IU; VE 18 IU; VK3 0.5 mg; nicotinic acid 9 mg; pantothenic acid 10 mg; biotin 0.09 mg; choline chloride 360 mg; copper 3 mg; zinc 50 mg; ferrum 60 mg; iodine 0.12 mg; manganese 2 mg; selenium 0.13 mg. Note ^2^: Digestible energy and lysine are calculated values (derived from nutrient content per ingredient in the nutrition facts table × ingredient ratio), while the remaining values are measured.

**Table 3:** Effect of compound probiotics on performance of pregnant sows.

Items	Group		
	Control Group	Experimental Group I	Experimental Group II
Piglet birth weight (kg)	1.39 ± 0.19 ^a^	1.25 ± 0.07 ^b^	1.19 ± 0.09 ^b^
Number of liveborn piglets	10.71 ± 1.10 ^b^	12.57 ± 0.57 ^a^	12.86 ± 1.04 ^a^
Number of healthy offspring	8.71 ± 1.25 ^c^	10.00 ± 0.76 ^a^	9.50 ± 1.03 ^b^
Litter birth weight (kg)	12.32 ± 0.68 ^c^	13.99 ± 0.82 ^a^	13.26 ± 0.35 ^b^
Weaning weight (kg)	7.82 ± 0.84	7.95 ± 0.35	8.02 ± 0.40
Weight of weaning litter (kg)	62.66 ± 3.68 ^b^	74.73 ± 2.75 ^a^	71.24 ± 2.02 ^a^

Note: In the same row, values with no letter or the same letter superscripts mean no significant difference (*p* > 0.05), while those with different small letter superscripts mean significant difference (*p* < 0.05).

**Table 4:** Colostrum immune indicators of lactating sows supplemented with compound probiotics.

Items	Group		
	Control Group	Experimental Group I	Experimental Group II
IgG (Immunoglobulin G) μg/mL	217.81 ± 5.77 ^c^	239.94 ± 6.03 ^a^	228.24 ± 4.33 ^b^
IgM (Immunoglobulin M) μg/mL	7.97 ± 0.44 ^c^	9.19 ± 0.37 ^b^	10.25 ± 0.20 ^a^
IgA (Immunoglobulin A) μg/mL	28.88 ± 3.12 ^b^	26.12 ± 2.37 ^c^	33.04 ± 1.42 ^a^

Note: In the same row, values with no letter or the same letter superscripts mean no significant difference (*p* > 0.05), while those with different small letter superscripts mean significant difference (*p* < 0.05).

**Table 5:** Effect of compound probiotics preparation on air index of pregnant sows.

Items	Group		
	Control Group	Experimental Group I	Experimental Group II
NH_3_ (Ammonia) mg/m^3^	19.73 ± 1.59 ^a^	14.44 ± 1.20 ^b^	12.92 ± 1.76 ^b^
H_2_S (Hydrogen Sulfide) mg/m^3^	1.82 ± 0.14	1.52 ± 0.19	1.50 ± 0.27
CO_2_ (Carbon Dioxide) mg/m^3^	1816.90 ± 42.90 ^a^	1748.60 ± 38.89 ^a^	1532.14 ± 39.28 ^b^
PM10 (Particulate Matter 10) μg/m^3^	55.00 ± 3.20	52.00 ± 1.87	54.00 ± 1.70
PM2.5 (Particulate Matter 2.5) μg/m^3^	39.00 ± 2.20 ^a^	32.00 ± 2.10 ^ab^	29.00 ± 1.40 ^b^
Humidity H %	64.00 ± 3.00	62.00 ± 1.00	64.00 ± 2.00

Note: In the same row, values with no letter or the same letter superscripts mean no significant difference (*p* > 0.05), while those with different small letter superscripts mean significant difference (*p* < 0.05).

**Table 6:** Growth Performance of Weaned Piglets supplemented with compound probiotics.

Items	Group			
	Control Group	Experimental Group I	Experimental Group II	Experimental Group III
IBW (Initial Body Weight)/kg	8.04 ± 0.59	7.99 ± 0.44	8.09 ± 0.56	7.85 ± 0.46
FBW (Final Body Weight)/kg	28.13 ± 0.15 ^b^	28.11 ± 0.25 ^b^	29.83 ± 0.35 ^a^	30.49 ± 0.31 ^a^
ADG (Average Daily Gain)/g	476.19 ± 3.55 ^b^	472.88 ± 4.48 ^b^	517.62 ± 6.62 ^a^	539.78 ± 5.72 ^a^
ADFI (Average Daily Feed Intake)/g	723.80 ± 18.23 ^b^	719.40 ± 9.87 ^b^	744.12 ± 10.25 ^a^	761.09 ± 13.45 ^a^
F/G (Feed/Gain)	1.52 ± 0.02 ^a^	1.46 ± 0.04 ^ab^	1.44 ± 0.04 ^bc^	1.40 ± 0.02 ^c^
Diarrhea rate (%)	7.52 ± 0.75 ^a^	6.75 ± 1.02 ^a^	5.40 ± 0.75 ^b^	4.50 ± 0.45 ^b^

Note: In the same row, values with no letter or the same letter superscripts mean no significant difference (*p* > 0.05), while those with different small letter superscripts mean significant difference (*p* < 0.05).

**Table 7:** Effect of compound probiotics on apparent digestibility of nutrients in weaned piglets.

Items	Group			
	Control Group	Experimental Group I	Experimental Group II	Experimental Group III
CP (Crude Protein)	76.46 ± 2. 19	78.24 ± 2.03	79.61 ± 1.45	81.03 ± 0.84
EE (Ether Extract)	73.16 ± 2.17	73.19 ± 4.09	75.90 ± 2.32	76.05 ± 2.79
CF (Crude Fiber)	49.35 ± 1.29 ^b^	53.16 ± 1.33 ^a^	54.05 ± 1.48 ^a^	55.25 ± 2.04 ^a^
GE (Gross Energy)	78.20 ± 1.12 ^b^	80.09 ± 1.08 ^b^	82.90 ± 0.24 ^a^	83.14 ± 1.24 ^a^
Ca (Calcium)	58.42 ± 1.74	60.05 ± 2.96	61.63 ± 2.16	62.15 ± 3.24

Note: In the same row, values with no letter or the same letter superscripts mean no significant difference (*p* > 0.05), while those with different small letter superscripts mean significant difference (*p* < 0.05).

**Table 8:** Effects of compound probiotics on serum biochemical indexes of weaned piglets.

Items	Group			
	Control Group	Experimental Group I	Experimental Group II	Experimental Group III
AKP (Alkaline Phosphatase) U/L	24.10 ± 2.08	22.49 ± 2.15	22.61 ± 1.03	22.75 ± 0.70
AST (Aspartate Transaminase) U/L	139.16 ± 6.64 ^b^	166.56 ± 3.34 ^a^	135.32 ± 4.38 ^b^	159.15 ± 7.64 ^a^
ALT (Alanine Aminotransferase) U/L	135.51 ± 1.79 ^a^	126.64 ± 1.45 ^ab^	110.36 ± 15.73 ^b^	128.64 ± 0.93 ^ab^
Ca (Calcium) mmol/L	3.35 ± 0.17 ^ab^	2.99 ± 0.20 ^bc^	3.56 ± 0.12 ^a^	3.17 ± 0.12 ^abc^
P (Phosphorus) mmol/L	1.64 ± 0.03 ^a^	1.45 ± 0.07 ^b^	1.69 ± 0.04 ^a^	1.71 ± 0.06 ^a^
BUN (Blood Urea Nitrogen) mmol/L	4.19 ± 0.05 ^a^	3.69 ± 0.58 ^ab^	3.39 ± 0.13 ^b^	3.45 ± 0.25 ^b^
ALB (Albumin) g/L	39.42 ± 0.36	44.35 ± 5.12	42.43 ± 1.92	43.36 ± 3.77
TP (Total Protein) g/L	58.77 ± 1.13	58.79 ± 1.63	60.32 ± 2.84	59.76 ± 1.18
T-CHOL (Total Cholesterol) mmol/L	0.32 ± 0.05	0.30 ± 0.04	0.30 ± 0.04	0.32 ± 0.02

Note: In the same row, values with no letter or the same letter superscripts mean no significant difference (*p* > 0.05), while those with different small letter superscripts mean significant difference (*p* < 0.05).

**Table 9:** Effects of compound probiotics on serum antioxidant indexes of weaned piglets.

Items	Group			
	Control Group	Experimental Group I	Experimental Group II	Experimental Group III
T-AOC (Total Antioxidant Capacity) U/L	2.34 ± 0.12	2.39 ± 0.21	2.44 ± 0.13	2.49 ± 0.05
MDA (Malondialdehyde) nmol/mL	3.90 ± 0.08	4.12 ± 0.34	3.65 ± 0.32	3.68 ± 0.29
SOD (Superoxide Dismutase) U/mL	59.75 ± 2.10 ^b^	63.24 ± 1.80 ^a^	63.76 ± 2.41 ^a^	66.24 ± 2.56 ^a^

Note: In the same row, values with no letter or the same letter superscripts mean no significant difference (*p* > 0.05), while those with different small letter superscripts mean significant difference (*p* < 0.05).

**Table 10:** Effect of compound probiotics on serum immune indexes of weaned piglets.

Items	Group			
	Control Group	Experimental Group I	Experimental Group II	Experimental Group III
IgG (Immunoglobulin G) μg/mL	121.18 ± 7.24 ^ab^	103.27 ± 3.96 ^b^	126.19 ± 8.51 ^ab^	129.11 ± 8.87 ^a^
IgM (Immunoglobulin M) μg/mL	8.86 ± 0.68	7.16 ± 0.36	7.83 ± 0.41	8.17 ± 0.45
IgA (Immunoglobulin A) μg/mL	24.02 ± 1.42 ^b^	23.77 ± 1.44 ^b^	25.52 ± 2.39 ^b^	28.01 ± 2.07 ^a^
IL-2 (Interleukin-2) pg/mL	745.63 ± 36.35	718.1 ± 66.35	737.71 ± 64.52	758.83 ± 70.16
IL-4 (Interleukin-4) pg/mL	301.21 ± 26.13	308.93 ± 25.41	317.25 ± 27.63	293.34 ± 29.13
C3 (Complement 3) pg/mL	6.59 ± 0.32	6.64 ± 0.42	7.27 ± 0.74	7.13 ± 0.43
C4 (Complement 4) pg/mL	69.97 ± 3.12	77.53 ± 3.45	76.25 ± 5.06	79.75 ± 4.59

Note: In the same row, values with no letter or the same letter superscripts mean no significant difference (*p* > 0.05), while those with different small letter superscripts mean significant difference (*p* < 0.05).

## Data Availability

The original contributions presented in this study are included in the article. Further inquiries can be directed to the corresponding authors.

## Abbreviations

The following abbreviations are used in this manuscript:

ADFI	Average Daily Feed Intake
ADG	Average Daily Gain
Lys	Lysine
F/G	Feed/Gain
CP	Crude Protein
EE	Ether Extract
DE	Digestible Energy
CF	Crude Fiber
CaHPO_4_	Calcium Hydrogen Phosphate
NaCl	Sodium Chloride
NH_3_	Ammonia
H_2_S	Hydrogen Sulfide
CO_2_	Carbon Dioxide
PM10	Particulate Matter 10
PM2.5	Particulate Matter 2.5
P	Phosphorus
Ca	Calcium
IBW	Initial Body Weight
FBW	Final Body Weight
GE	Gross Energy
AKP	Alkaline Phosphatase
ALB	Albumin
TP	Total Protein
ALT	Alanine Aminotransferase
AST	Aspartate Transaminase
BUN	Blood Urea Nitrogen
T-CHOL	Total Cholesterol
T-AOC	Total Antioxidant Capacity
MDA	Malondialdehyde
SOD	Superoxide Dismutase
IgG	Immunoglobulin G
IgM	Immunoglobulin M
IgA	Immunoglobulin A
IL-2	Interleukin-2
IL-4	Interleukin-4
C3	Complement 3
C4	Complement 4
